# Volume XVI

**Published:** 1878-04

**Authors:** 


### Editorial.

### VOLUME XVI.

With this number we commence the sixteenth volume of the JOURNAL, and by the additional expense in the purchase of new type and a better quality of paper, seek to render it more attractive than heretofore. While this attention has been bestowed upon the dress, we will endeavor not to lose sight of the fact that in the make-up of a journal, the selection and arrangement of reading matter are still more important to success. The great object of periodicals is to afford regularly the reports of improvements and discoveries being made throughout the world. In this way individual members of the profession are enabled to keep pace with the advancement of science, and reap advantages therefrom in their efforts to combat disease.

Notwithstanding the imperative demands upon our time and attention, incident to the regular practice of medicine, exchanges and monographs are consulted in order to afford the latest and best items of interest. We may not be a successful caterer and always please, but our labors are directed toward the advancement of medical knowledge. We are gratified in the reflection that we have so far succeeded in our efforts as to secure paying patronage that will authorize continuance of the publication. Several Southern journals have succumbed to the pecuniary depression resulting from war's devastation, and if full compensation for labor in dollars and cents had been the sole object of continuance, this also would have ceased to exist.

By our efforts to improve the JOURNAL, we hope to inspire increased interest on the part of its patrons, and extend the circulation to other paying subscribers. Though pecuniary embarrassment pervades the whole country, and individuals—doctors especially—find their hard-earned dollars coming in slowly, yet the small annual sum from each subscriber required to keep the JOURNAL such as they wish and need, will scarcely be felt. None feel the loss until, by neglecting to pay year after year, the amount increases so as to become rather embarrassing to those with an income barely sufficient to pay yearly expenses.

